# COVID-19: What we’ve done well and what we could or should have done better—the 4 Ps

**DOI:** 10.1186/s13054-021-03467-y

**Published:** 2021-01-28

**Authors:** Jean-Louis Vincent, Julia Wendon, Greg S. Martin, Nicole P. Juffermans, Jacques Creteur, Maurizio Cecconi

**Affiliations:** 1 Department of Intensive Care, Erasme Hospital, Université Libre de Bruxelles, Route de Lennik 808, 1070 Brussels, Belgium; 2 Department of Critical Care, Kings College Hospital Foundation Trust, London, UK; 3 Division of Pulmonary, Allergy, Critical Care and Sleep Medicine, Department of Medicine, Emory University School of Medicine, Atlanta, GA USA; 4 Laboratory of Experimental Intensive Care and Anesthesiology, Amsterdam University Medical Centre, Location Academic Medical Centre, Amsterdam, The Netherlands; 5 Department of Intensive Care, OLVG Hospital, Amsterdam, The Netherlands; 6 Department of Anesthesiology and Intensive Care, Humanitas Clinical and Research Center-IRCCS, Rozzano, Milan Italy; 7 Department of Biomedical Sciences, Humanitas University, Pieve Emanuele, Milan, Italy

**Keywords:** Coronavirus, Personalization, Clinical trial design, Sepsis, ICU organization, Endotheliopathy, Acute respiratory failure, ICU admission criteria, Ethical decision making

## Abstract

The current coronavirus pandemic has impacted heavily on ICUs worldwide. Although many hospitals and healthcare systems had plans in place to manage multiple casualties as a result of major natural disasters or accidents, there was insufficient preparation for the sudden, massive influx of severely ill patients with COVID-19. As a result, systems and staff were placed under immense pressure as everyone tried to optimize patient management. As the pandemic continues, we must apply what we have learned about our response, both good and bad, to improve organization and thus patient care in the future.

*“Study the past if you would define the future” (Confucius)*

## Introduction

Since the SARS-CoV-2 pandemic first began, strategies to minimize spread of the virus in the community have been employed but often not early, extensive or effective enough to prevent multiple hospitalizations, intensive care unit (ICU) admissions and deaths. Thus, as the pandemic continues, we, as intensivists, need to be constantly evaluating and reevaluating our response, to review what we have learned, what we have accomplished so far, what we have done well and, perhaps more importantly, what we could have done better.

Here we do just that, dividing our thoughts into four broad areas, the 4 Ps: Preparation, Progress, Personalization, and Prioritization (Table 1). This reflection can help us optimize patient care and ICU organization as coronavirus disease (COVID-19) continues to affect thousands of people worldwide, and will also help as we plan for the likely occurrence of similar situations in the future.

**Table 1:** Some of the aspects we have done well and some we could have done better

	Did well	Could have done better
Preparation	Using other areas of the hospital to expand ICU capacity Distributing resources	Increasing resources when still in pre-epidemic period Providing more psychological support in some centers
Progress	Understanding the pathophysiology Improving general, especially respiratory, management Discovering benefit from corticosteroid administration	Increasing international collaboration Increasing definitive RCTs on therapeutic interventions in critically ill patients Decreasing enthusiasm for unproven and theoretical treatments Increasing research on susceptibility and host response factors
Personalization	Rapidly deciphering individual responses and patterns of disease	Individualizing ARDS management Paying more attention to tissue perfusion Making greater use of biomarkers to guide therapy
Prioritization	Developing guidelines	Discussing ethical uncertainties and optimizing ICU admissions in some centers Coordinating resources across centers

## Preparation

As we began to realize the likely impact of the pandemic on our healthcare systems and our ICUs in particular, we rapidly began to prepare by converting other areas of the hospital, such as operating rooms and anesthesia recovery units, into temporary ICUs. Although this was necessary given the large numbers of patients requiring intensive care, it was often done at the expense of routine, non-urgent procedures, which likely has led to increased *non-*COVID morbidity and mortality, the magnitude of which is only beginning to be fully assessed. Available resources were distributed as evenly as possible among units and hospitals to ensure adequate supplies for all [1]. Nurses and doctors, including those still in training, were transferred from other units to work on the ICU, although training of such personnel was often rushed and inadequate. To enhance training, several groups and societies contributed to rapid expansion of online education, including the European Commission and the European Society of Intensive Care Medicine (ESICM) C19_SPACE program (COVID-19 Skills Preparation Course) developed to help provide better training for healthcare workers not working regularly in intensive care [2], and the Society for Critical Care Medicine’s (SCCM) training for non-ICU clinicians [3].

Using imagination and innovation, new, simple continuous positive airway pressure (CPAP) systems and basic respirators (even car factories started to make some) were developed and constructed, to meet the increased demand for respiratory support. New methods of involving and informing relatives, who were not allowed to visit their loved ones, and of limiting the feelings of loneliness for patients were devised, including employing video calls.

Of course, with hindsight we could, and indeed should, have been better prepared. Methods to ensure sufficient adequately trained personnel, enough appropriately equipped ICU beds, and adequate amounts of material -from personal protective equipment to ventilators- could all have been better planned and potential alternative supply chains already identified. Plans should have been established to enable transport of patients to hospitals in areas of the country that were less badly affected, or even across international borders. Many countries now have a central distribution system in place ensuring an even spread of patients. Importantly, the provision of on-going psychological support for all those involved in the COVID pandemic -staff, patients and families- should have been foreseen and readily available, personalized to individual requirements.

## Progress

We have made substantial progress in our understanding of the pathophysiological alterations associated with COVID-19, particularly that the disease is not limited to the respiratory tract and altered lung function, but affects all organs. Identification of the associated endotheliopathy and coagulopathy [4] and documentation of the virus in virtually all organs [5] have helped us appreciate that the whole body is involved and not just the lungs. As a result, management has improved substantially with less use of invasive mechanical ventilation and more effective thrombosis prophylaxis. Mortality rates have also decreased, likely as a result of the improved understanding of the disease process and better patient management [6].

However, we may have made more progress in specific treatments if we had explored the effects of (old and) new therapeutic interventions more carefully. With pressure to identify effective treatments and the feeling that something had to be done to help save lives, we tried too many drugs without testing them rigorously. Results from RECOVERY, SOLIDARITY and REMAP-CAP adaptive research platforms have since demonstrated that many of our initial assumptions were wrong and did not bring any benefit [7, 8]. One example is in the early studies on hydroxychloroquine, results of which were published online without going through the usual rigorous, peer review process. The resultant initial enthusiasm for hydroxychloroquine, with its associated media hype and support from leading celebrities and political figures, became an obstacle to performing effective randomized controlled trials of the drug because high patient and family demand for it to be given limited our ability to randomize patients. This publicity also hampered trials assessing other interventions, because of the concern that the use of hydroxychloroquine could add noise to the trial. Another example is the drug remdesivir. Medical and pharmacoeconomic models were massively involved in propagating a therapy that was later shown not to be very effective, at least in severely ill patients [7]. As with many other aspects of management of this pandemic, international collaboration for clinical trials was poor and results could have been improved by using better structured, international platforms already early in the pandemic. Identification of the benefits of corticosteroid administration was one example of a well-run clinical trial [9]. However, as a result of early publication of preliminary results from the RECOVERY trial, other ongoing trials were stopped early, limiting full interpretation of the results from these later trials [10]. Importantly too, there are many other interventions that could and should be tested. For example, the endotheliopathy/coagulopathy may represent an excellent indication for substances like thrombomodulin [11]. There has also not been enough research on factors that influence susceptibility to severe manifestations of COVID-19 and the host response [12], although this may still come.

## Personalization

COVID-19 is a single disease, but individual responses can vary from one person to another and over time, making it a more heterogeneous condition than initially thought. At the start of the pandemic, a lot of attention was paid to the development of acute respiratory distress syndrome (ARDS) in patients with COVID-19, with standardized respirator management being given to *all* patients with acute respiratory failure, before we realized the respiratory failure can be multifaceted in these patients, with some having more focal alterations [13]. Indeed, although ARDS is a common complication of COVID-19, it is often not severe initially. Nevertheless, early in the pandemic, patients sometimes received excessive positive end-expiratory pressure (PEEP) based on the severity of alteration in gas exchange, but with lung alterations that were not diffuse. Perhaps we spent too much time trying to find differences between COVID ARDS and non-COVID ARDS, and global differences in thoraco-pulmonary compliance whereas treatment should be individualized according to each patient’s specific respiratory status. Initially too, a focus on avoiding lung edema led to restrictive fluid therapy with liberal use of diuretics, when in fact some patients were hypovolemic, leading to a high incidence of mesenteric ischemia and renal injury, and may have benefitted from fluid administration. Not enough attention was paid to the alterations in tissue perfusion that require individual monitoring and management. We understood that treatment of early COVID-19 was likely to be different from that of later stage disease, but essentially related this to the severity of the disease and the degree of alteration in gas exchange.

As with other areas of critical illness, management of patients with COVID-19 needs to be personalized to the individual patient. In general terms, treatment is biphasic, with a focus on antiviral therapies initially, but immunomodulating strategies in the later phase, when viral replication is no longer the major issue. As such, we could have better used biomarkers [14] to identify an excessive host response, which has sometimes been (mis)called a ‘cytokine storm’ [15]. Substances such as tocilizumab and anakinra may be effective in some subsets of patients, whereas benefits may not appear in heterogeneous groups of patients who have not been carefully selected. Similarly, better understanding of how to determine the optimal dose of anticoagulation is needed so that it can be personalized for individual patients.

## Prioritization

The substantial and unexpectedly rapid surge in numbers of SARS-CoV-2 infected patients needing ICU admission meant that rapid decisions frequently had to be made regarding suitability for admission to critical care. Decision making in this scenario was based largely on the presence of comorbidities and taking into account patient preferences. Although age has been widely reported as among the factors influencing decisions regarding ICU admission, frailty and life expectancy are more important than age. Despite publication of guidelines [16–19], the ways in which units and individuals interpreted the basic principles varied considerably, often leading to confusion and distress. To avoid subjective assessments and inequities, the principle of a lottery or a ‘first come, first served’ principle was sometimes considered [20]. When critical resources, such as ICU beds and respiratory support modalities, can be effectively expanded, this is of course preferable; but, if all critical care resources are fully occupied by patients with a poor prognosis who are less likely to benefit from intensive care, the resources can no longer be used to provide life-saving care to other patients with a greater chance of recovery. The key in such decisions is to identify the patients who will benefit most, based on the combination of their physiological reserve, the status of their acute illness, and their response to therapy. Difficult decisions about stopping life-sustaining therapies when patients are continuing to deteriorate despite optimal interventions also have to be reviewed and should be assessed in the context of physiology, background health and appropriate ethical frameworks. These decisions are not just related to the pandemic. Intensivists regularly need to make such decisions to ensure intensive care need and supply are balanced. Carefully defined criteria for ICU admissions and for withholding and withdrawing life-sustaining therapies should therefore be in place at all times.

Use of interventions that demand a high level of care, such as extracorporeal membrane oxygenation (ECMO), may also need reconsidering when resources are stretched. Provision of resource intense therapies to a limited number of patients may need to be balanced against the provision of less advanced care to more patients [18]. Importantly, care for other conditions requiring ICU capacity must continue for patients presenting as emergencies and also for those requiring complex elective surgery, whose life expectancy will be markedly impacted if there is any significant delay in their treatment. We also need to address the streaming of patients into COVID-positive, emergency and elective care to provide optimal provision and limit nosocomial spread. Prioritization thus applies to COVID and non-COVID patients equally.

Importantly, we have had to learn to accept that, under such demanding conditions and with limited resources (in terms of number and quality), it is not always possible or practical to offer the quality of care we would wish and are used to providing; this is very difficult to accept psychologically, but is the only way to manage the increased patient numbers.

## Conclusion

As we continue to move through this pandemic, with many countries still challenged by high case numbers and hospitalizations, these aspects of preparation, progress, personalization and prioritization will continue to be discussed and our approach must evolve according to new discoveries and changes in pandemic presentation and patient demographics. There is already much we have learned that will improve the way in which we face the rising numbers of admissions and manage our patients. What we have learned and will learn over the next weeks and months will help us prepare for similar events in the future, will fuel continued progress in disease understanding and management, will encourage us to personalize patient treatment, and will facilitate the difficult decisions to prioritize care when necessary.

## Data Availability

Not applicable.

## Abbreviations

ARDS: Acute respiratory distress syndrome
CPAP: Continuous positive airway pressure
ECMO: Extracorporeal membrane oxygenation
ESICM: European Society of Intensive Care Medicine
ICU: Intensive care unit
PEEP: Positive end-expiratory pressure
SCCM: Society of Critical Care Medicine
